# Contribution of perceived loneliness to suicidal thoughts among French university students during the COVID-19 pandemic

**DOI:** 10.1038/s41598-022-21288-z

**Published:** 2022-10-07

**Authors:** Melissa Macalli, Shérazade Kinouani, Nathalie Texier, Stéphane Schück, Christophe Tzourio

**Affiliations:** 1 University of Bordeaux, Inserm, Bordeaux Population Health Research Center, UMR 1219, F-33000 Bordeaux, France; 2 Kappa Santé, 4 Rue de Cléry, 75002 Paris, France

**Keywords:** Psychology, Risk factors

## Abstract

Restrictive measures during the COVID-19 epidemic have led to increased levels of loneliness, especially among university students, although the influence on suicidal thoughts remains unclear. In this cross-sectional study of 1913 French university students, those with the highest level of loneliness had a fourfold increased risk of suicidal thoughts. Perceived loneliness should be incorporated into suicide risk assessment, and assistance in coping with loneliness should be considered as a means of reducing suicidal risk in vulnerable groups, like university students.

## Introduction

Loneliness is defined as a subjective experience where one perceives a discrepancy between their desired and current levels of social relationships^1,2^. The negative effects of loneliness on mental health are well documented^3–6^. In particular, loneliness has been associated with increased risk of engaging in suicidal behaviors, supported by theoretical models of suicide, such as Joiner’s interpersonal theory of suicide^7^. According to this theory, suicidal desire is caused by the simultaneous presence of two interpersonal constructs: thwarted belongingness and perceived burdensomeness. Thwarted belongingness includes loneliness and the absence of reciprocal care. Self-reported loneliness is considered as one of the six observable risk factors described by Joiner to measure loneliness^7^.

University students are particularly exposed to suicidal behaviors and psychiatric disorders which often start in young adulthood^8–10^. Emerging adulthood is already a specific period of multiple changes^11^ and university life implies adaptation to a new environment. Factors that may contribute to an increased risk of mental health disorders and suicidal behaviors in this population include the transition from high-school to university, increasing workload and psychosocial stress or academic pressure^12^. Thus, before the COVID-19 pandemic, college students already had a high prevalence of mental health problems^13–15^.

In response to the pandemic, numerous countries implemented restrictions, such as lockdowns, curfews, physical distancing requirements, and university closures. This context affected social support and limited personal interactions, while increasing the intensity of several stressors especially among the vulnerable population^16,17^. Some recent studies have revealed that, due to the social-distancing measures, loneliness became a predominant determinant for anxiety, depression, and their comorbidity^18,19^. These restrictions have increased levels of loneliness among the general population including university students during the COVID-19 pandemic^20,21^, which could have negatively impacted their mental health^22,23^.

A recent study showed that loneliness was consistently associated with the incidence of suicidal ideation, while other objectives measures of social relationship during the COVID-19 outbreak, such as living alone, or the number of days practicing social distancing, were not^24^. However, information on the association between perceived loneliness and suicidality among university students in this epidemic context is scarce. The objectives of this study were to measure the contribution of perceived loneliness to suicidal ideation among French college students during the COVID-19 pandemic, and to explore the role of depressive symptoms in this association.

## Methods

### Design, study population, and data source

Our study sample comprised participants in the web-based CONFINS cohort (www.confins.org), a prospective population-based study launched in March 2020, at the very beginning of the first lockdown in France, to address the psychological impact of COVID-19. Participants were recruited through advertisements on traditional and social media. Eligible university students had to be ≥ 18 years of age, and to have been confined in France. In this cross-sectional study, baseline data were collected between March 2020 and January 2021. Over this period, data collection occurred during different key periods of the COVID-19 epidemic in France.

### Measures

Perceived loneliness was investigated with the question: “In the last seven days, on a scale of 0–10 (0 = not at all, 10 = totally), how lonely do you feel?” We decided to compare students with high versus low levels of loneliness. For this purpose, we compared the group of students in the highest quartile of the scale (score ≥ 7) (later labeled “loneliness”) to the remaining students (labeled “no loneliness”). The questionnaire included a single-item question about suicidal thoughts during the last seven days: “In the last seven days, how often have you thought of attempting suicide (had suicidal ideation)?” Participants selected one of three responses: no suicidal thoughts, occasional suicidal thoughts, or frequent suicidal thoughts. In the present analysis, occasional and frequent suicidal thoughts were grouped together. The analyses included the following covariates as potential confounders: age, gender (male, female, other), marital status (single, married, in a couple), inclusion period (first national lockdown: March 17–May 11, 2020; no lockdown restrictions: May 12–October 27, 2020; second national lockdown and curfew: October 28, 2020–January 25, 2021), social interactions by phone (never, less than once weekly, more than once weekly), social interactions on social networks (never, less than once weekly, more than once weekly), history of psychiatric disorders (yes, no), and depressive symptoms measured with the 9-item Patient Health Questionnaire (PHQ-9)^25,26^ using a cut-off of 10 to define the presence of depressive symptoms. Participants with a PHQ-9 score ≥ 10 were further later labeled “with depressive symptoms” and the others were labeled “without depressive symptoms”^27^.

### Statistical analyses

We used binary logistic regression models to measure the association between loneliness and suicidal thoughts. To explore whether the effect of loneliness on the risk of suicidal thoughts was independent of depressive symptoms, we repeated analyses in the two strata with and without depressive symptoms defined above. Finally, we estimated the contribution of loneliness to suicidal thoughts by calculating its attributable fraction (AF), which is the proportion of suicidal thoughts attributable to loneliness, calculated as follows: AF = [p*(aOR − 1)]/[1 + (p*(aOR − 1)], where p represents the prevalence of loneliness in our study sample, and aOR represents the adjusted odds ratio related to the association between exposure and the outcome in multivariate models. All analyses were performed using SAS version 9.4. Two-sided *p* values of < 0.05 were considered statistically significant.

### Ethical approval and consent to participate

The study follows the principles of the Declaration of Helsinki, and the data collection, storage, and analysis comply with the General Data Protection Regulation (EU GDPR). The study was approved by the French Committee for the Protection of Individuals (Comité de Protection des Personnes – CPP IDF X, nr. 46-2020) and by the National Commission on Informatics and Liberty (Commission Nationale de l'Informatique et des Libertes) CNIL, nr. MLD/MFI/AR205600). Participants were informed of the nature and purpose of the study, and provided online consent. Informed consent was obtained from all subjects.

## Results

The study population included 1913 college students. The mean age was 23.5 years (± 3.8), and 80.7% were female (N = 1544). Loneliness was reported by 26.9% of participants (N = 514). The prevalence of suicidal thoughts was 12.7% (N = 242) including occasional (10.3%; N = 197) or frequent (2.35%; N = 45). Students with loneliness reported significantly more suicidal thoughts (27.2% vs 7.3%; *p* < 0.0001), depressive symptoms (67.1% vs 26.1%; *p* < 0.0001), and history of psychiatric disorders (29.6% vs 18.6%; *p* < 0.0001) compared to students without loneliness (Table 1). During the second lockdown, the proportion of students who reported loneliness was significantly higher than those who did not (22.6% vs 10.4%; *p* < 0.0001).

**Table 1:** Study sample characteristics within the whole sample and according to loneliness.

	Total sample (N = 1913)	Loneliness (N = 514; 26.9%)	No loneliness (N = 1399; 73.1%)
**Age**, Mean (SD)	23.5 (3.8)	23.2 (4.1)	23.6 (3.7)
**Female**	1544 (80.7)	415 (80.7)	1129 (80.7)
**In couple/married**	917 (47.9)	169 (32.8)	748 (53.5)
**Suicidal thoughts**	242 (12.7)	140 (27.2)	102 (7.3)
**Depressive symptoms**	710 (37.1)	345 (67.1)	365 (26.1)
**History of psychiatric disorders**^**a**^	397 (21.5)	144 (29.6)	253 (18.6)
**Inclusion period**			
First lockdown	1194 (62.4)	311 (60.5)	883 (63.1)
No lockdown	458 (23.9)	87 (16.9)	371 (26.5)
Second lockdown/Curfew	261 (13.6)	116 (22.6)	145 (10.4)

All data presented as n (%), unless otherwise noted.

*SD* standard deviation.

^a^Data missing for 67 participants.

Adjusted logistic regression models revealed a strong association between loneliness and suicidal thoughts (Table 2). Loneliness was associated with a fourfold higher risk of suicidal thoughts (aOR = 4.34; 95% CI: 3.17–5.95), with adjustment for age, gender, inclusion period, interactions by phone, interactions on social network, marital status, and history of psychiatric disorders. In analyses stratified for depressive symptoms, this association decreased but remained high, and in the same proportion regardless of whether the participants reported depressive symptoms. Thus, students who reported loneliness were at higher risk of suicidal thoughts with depressive symptoms (aOR = 2.44; 95% CI: 1.68–3.56) or without depressive symptoms (aOR = 3.76; 95% CI:1.43–8.12) (Table 3). Finally, we observed that the AF of suicidal thoughts imputed to loneliness was high within the total study sample (47.3%), as well as among students with depressive symptoms (41.2%) or without (28.0%) (Table 3).

**Table 2:** Association between loneliness and suicidal thoughts.

	**aOR**	**95% CI**	***P*-value**
**Main exposure**			
**Loneliness**			**< 0.0001**
No	Ref		
Yes	4.34	[3.17–5.95]	
**Covariates**			
**Age**	0.95	[0.91–1.00]	**0.035**
Gender			
Male	Ref		0.372
Female	0.98	[0.65–1.46]	
Other	2.30	[0.66–8.02]	
**Inclusion period**			0.680
No lockdown	Ref		
First lockdown	0.85	[0.58–1.24]	
Second lockdown/Curfew	0.93	[0.57–1.53]	
**Interactions by phone**			0.848
More than once weekly	Ref		
Less than once weekly	0.99	[0.63–1.55]	
Never	1.18	[0.66–2.11]	
**Interactions on social network**			0.404
More than once weekly	Ref		
Less than once weekly	1.31	[0.75–2.29]	
Never	1.35	[0.77–2.34]	
**Marital status**			0.429
Married/in couple	Ref		
Single	0.88	[0.65–1.21]	
**History of psychiatric disorders**			**< 0.0001**
No	Ref		
Yes	3.93	[2.87–5.38]	

Binary logistic regression models.

Adjusted on age, gender, inclusion period, interactions by phone, interactions on social network, marital status and history of psychiatric disorders.

Significant values are in bold.

**Table 3:** Association between loneliness and suicidal thoughts in the total sample and categorized by whether individuals had depressive symptoms.

	Total			Depressive symptoms					
				With			Without		
	aOR	95% CI	AF(%)	aOR	95% CI	AF(%)	aOR	95% CI	AF(%)
Loneliness	4.34	[3.17–5.95]	47.3	2.44	[1.68–3.56]	41.2	3.76	[1.43–8.12]	28.0

Binary logistic regression models and attributable fractions.

Adjusted on age, gender, inclusion period, interactions by phone, interactions on social network, marital status and history of psychiatric disorders.

*AF* attributable fraction.

## Discussion

In this sample of French university students enrolled during the COVID-19 epidemic, we found that loneliness was associated with a fourfold increased risk of suicidal thoughts (aOR = 4.34; 95% CI: 3.17–5.95), independently of major potential confounders, e.g., social interactions or history of psychiatric disorders. About half of suicidal thoughts (47.3%) could be attributed to loneliness in this population.

Loneliness has been found to be associated with suicidal behavior in the general population^28,29^ and particularly so during the COVID-19 pandemic^18,19^ but few studies were performed on the contribution of loneliness to suicidal thoughts among students during the COVID-19 pandemic. The high estimates, and especially the high AFs, we found, confirm the importance of this risk factor in the students population.

Although lonely individuals are more vulnerable to depression^30,31^, we found that loneliness remained strongly associated with suicidal thoughts regardless of the presence of depressive symptoms. Analyses stratified on depressive symptoms showed that the association was statistically significant in both stratum and that the estimate was even higher among students without depressive symptoms (aOR = 3.76; 95% CI: 1.43–8.12) than in those with (aOR = 2.44; 95% CI: 1.68–3.56). These results suggest that loneliness could have an impact on suicidal thoughts independently of depressive symptoms. In the literature, the role of psychiatric disorders, especially depressive symptoms, on this association is unclear. In some studies loneliness was associated with suicidal thoughts independently of depression^32^, while in others this relationship was fully^33^ or partially mediated by depression^28^.

While we were not able to compare the levels of loneliness before and during the pandemic, we found that the highest level of loneliness was frequent in our study sample (more than one quarter of the sample) and among students who reported depressive symptoms (48.6%, data not shown), which explained the large AF for suicidal thoughts in these groups. One recent study showed that loneliness levels were higher during the COVID-19 pandemic than before^34^ and another showed that feelings of loneliness significantly increased during the pandemic among students^35^. The social isolation imposed from the beginning of the pandemic would have directly contributed to loneliness^36^. Young adults already reported high levels of loneliness^37,38^ and, because of the lockdowns and the systematic use of online courses and virtual communication, this feeling has increased during the pandemic^39^. This could also have had important consequences on their academic success, as loneliness may partially mediate the trajectory of mental health outcomes on academic performance^40^.

Our results should be interpreted in the light of the following limitations. First, since our study was cross-sectional, we could not strictly separate the timing of exposure, outcome, and covariates, and no causality can be inferred between exposure and suicidal thoughts. Longitudinal studies are needed to study this temporal relationship, the importance of loneliness in suicide prediction, and the potential role of depressive symptoms in mediating this relationship. Second, due to the potential self-selection and the over-representation of women in our sample of volunteers, our conclusions cannot be generalized to a wider population. Third, we measured suicidal thoughts by a single item. Since the CONFINS study covers several themes, it was not possible to use a full questionnaire compatible with an acceptable completion time and participation. Therefore, we chose to ask the question with only a single item adapted to young adult population after team’s consensus between psychiatrists and psychologists. This is a common approach, used in most population-based cohort studies^41,42^. Likewise, we measured loneliness using a self-reported single-item question that may fail to capture participants who under-report loneliness due to stigma^43^. However, loneliness assessment using a single-item question and a multiple-item scale reportedly show a high level of concordance^44^. Fourth, the self-reported questionnaires could lead to information bias, particularly if participants under-reported their frequency of suicidal thoughts or history of psychiatric disorders due to concerns about social desirability. Fifth, although we included several major covariates in our multivariate models, unmeasured confounding factors may remain and could partly explain the observed association.

If confirmed, our present results have two potentially important implications. First, in terms of population health, they show that loneliness is an important risk factor for suicidal thoughts among college students, and that interventions targeting loneliness may reduce suicidal risk in the context of aggravated social isolation (e.g., epidemic)^45^. Future approaches may include community initiatives and prescription of social activities, particularly among vulnerable groups, such as university students. As loneliness is increasingly recognized as a worldwide public health issue related to emerging societal trends^37^, the questions raised will remain of interest even beyond the context of an epidemic. Second, our results also suggest that perceived loneliness should be incorporated into suicide risk assessment. This question is shorter and less intrusive than questions regarding depressive symptoms or social interactions. This simple marker could be useful during pandemic periods, for stratifying groups and detecting individuals with a higher suicidal risk.

## Data Availability

The datasets used and/or analyzed during the current study are available from the corresponding authors on reasonable request.
